# Editorial: Emerging roles of miRNAs in cardiovascular disease

**DOI:** 10.3389/fcvm.2023.1144849

**Published:** 2023-02-28

**Authors:** Donato Santovito, Yuhua Fan, Leonardo Elia, Joanne T. M. Tan, Emiel P. C. van der Vorst

**Affiliations:** ^1^ Institute for Cardiovascular Prevention (IPEK), Ludwig-Maximillians-Universität (LMU), Munich, Germany; ^2^ German Center for Cardiovascular Research (DZHK), Partner Site Munich Heart Alliance, Munich, Germany; ^3^ Institute for Genetic and Biomedical Research, Unit of Milan, National Research Council, Milan, Italy; ^4^ Department of Basic Medical College, Harbin Medical University (Daqing), Daqing, China; ^5^ IRCCS Humanitas Research Hospital, Rozzano, MI, Italy; ^6^ Department of Molecular and Translational Medicine, University of Brescia, Brescia, Italy; ^7^ Vascular Research Centre, Lifelong Health Theme, South Australian Health and Medical Research Institute, Adelaide, SA, Australia; ^8^ Adelaide Medical School, The University of Adelaide, Adelaide, SA, Australia; ^9^ Aachen-Maastricht Institute for CardioRenal Disease (AMICARE), Interdisciplinary Center for Clinical Research (IZKF) and Institute for Molecular Cardiovascular Research (IMCAR), RWTH Aachen University, Aachen, Germany

**Keywords:** miRNA, cardiovascular disease, macrophages, biomarker, acute coronary syndrome

**Editorial on the Research Topic** Emerging roles of miRNAs in cardiovascular disease

MicroRNAs (miRNAs) are short sequences of single-stranded non-coding RNAs involved in RNA-dependent gene silencing by directing the RNA-induced silencing complex (RISC) toward target messenger RNAs to promote their translational repression or decay (1). Since their original discovery in *C. elegans*, hundreds of conserved miRNAs have been identified, and their contribution to regulating genes that are crucially involved in cell biology is supported by strong mechanistic evidence (1). As such, miRNAs feature key contributions in developmental and homeostatic processes, as well as in the pathogenesis of several diseases (1, 2). In particular, miRNAs are crucial for homeostasis and function of the cardiovascular system, and genetic deletion of *Dicer1*, encoding for the rate-limiting enzyme for miRNA biosynthesis, in mice inhibits angiogenesis and results in embryonic lethality (3). Similarly, the postnatal inhibition of the miRNA biosynthesis pathway either by conditional deletion of *Dicer1* in cardiomyocytes or vascular cells or by blocking proper RISC assembly results in failure of the cardiovascular function with the development of pathological phenotypes (i.e., dilatative cardiomyopathy or susceptibility to atherosclerosis) (4). Fueled by these observations, multiple studies have already investigated the role of miRNAs in cardiovascular disease and identified feasible therapeutic opportunities moving toward clinical translation, as shown by the completion of recent phase I clinical trials on inhibitors of miR-132-3p and miR-92a-3p for the treatment of heart failure (2, 5, 6). Yet, knowledge about the exact role of miRNAs in cardiovascular disease is far from reaching its completeness, and evidence from the last few years has also highlighted the relevance of unconventional mechanisms of miRNA functions, as well as their contribution to paracrine/endocrine cell communication upon release in the extracellular space, and their potential relevance as a biomarker of cardiovascular disease with potential clinical applications (7–10). The articles in this Research Topic further extend our knowledge on the roles and possible translational applications of miRNAs in cardiovascular disease.

Macrophages play important roles in cardiovascular disease. For example, macrophages are involved already in the early phases of atherogenesis, when they accrue modified lipoproteins and differentiate into foam cells, one of the hallmarks of atherosclerosis (11). However, macrophages are characterized by high phenotypical plasticity and, while they can perpetuate inflammatory responses to favor atheroprogression, their alternative activation may result in protective phenotypes (12). Among them, higher expression of ATP-binding cassette transporter A1 (ABCA1) prevents the formation of foam cells by aiding cholesterol efflux and reverse cholesterol transport, thus exerting protective roles against atherosclerosis (12). The *ABCA1* transcript is characterized by a remarkably long 3'UTR (>3.3 Kb) that binds to several miRNAs. Mechanistic studies as well as correlative analyses in humans revealed the importance of miRNAs in regulating ABCA1 (e.g., miR-33, miR-144, miR-758, and others) (13–15), and Aranda et al. further strengthen this evidence by showing the contribution of miR-199a-5p in regulating ABCA1 and cholesterol efflux in macrophages. Notably, miR-199a-5p is downregulated by hypoxia and hyperlipidemia, possibly implying the involvement of this pathway in cardiovascular pathophysiology, where cholesterol alterations and hypoxia play important roles.

Besides their intracellular regulatory role, miRNAs can be packaged for release in the extracellular space within vesicles (e.g., exosomes, microparticles), plasma lipoproteins, or associated with proteins (e.g., AGO2) (2, 10, 16). Notably, miRNAs can be reliably detected in most biological fluids (e.g., plasma, serum, urines) and studies have identified changes in their expression pattern with disease, highlighting their prospective use as diagnostic biomarkers (2, 9, 10). In their research article, Gager et al*.* report the results of a monocentric prospective study revealing a negative association between circulating miR-125b and all-cause mortality in patients with acute coronary syndrome (ACS). Moreover, circulating miR-125b was lower in patients suffering from ST-elevation ACS and directly correlated with plasma levels of inflammatory biomarkers (i.e., C-reactive protein). Furthermore, Venugopal et al. aimed to prioritize the choice of miRNAs as biomarkers for patients with ACS. In their study, they explored two already available datasets and integrated the data with algorithms to predict affected target genes and pathways. Their analyses identified four miRNAs (i.e., let-7b-5p, let-7c-5p, miR-342-3p, and miR-4505) as possible biomarkers for ACS, with involvement in pathways relevant to response to ischemia and cardiac disease, to be validated and tested in future prospective studies. While these findings will require independent replication in multicenter prospective clinical trials and while the prompt introduction of circulating miRNAs as biomarkers in clinical practice is still limited by multiple aspects (e.g., lack of universal consensus in the analytical/normalization approach) (2), these studies open up new avenues for future research with the ultimate endeavor of identifying new biomarkers for a better risk assessment and management of patients with ACS.

Finally, the Research Topic includes two critical review articles summarizing the current evidence on the role of miRNAs as biomarkers of site-specific atherosclerosis and pulmonary artery hypertension (PAH). Indeed, Teixeira et al*.* review the circulating miRNAs associated with atherosclerosis in distinct arterial districts, specifically aiming to identify a common circulating signature associated with a stable atherosclerotic phenotype independent of the affected artery. Moreover, Rogula et al. summarize the evidence on the possible applications of circulating miRNAs as diagnostic tools for distinguishing the different etiologies of PAH and as prognostic markers of disease severity. Finally, they conclude their overview by discussing miRNAs as potential therapeutic targets in PAH.

In conclusion, this Research Topic aims to provide a standpoint for further research focusing on miRNAs with the ultimate goal of identifying new diagnostic and prognostic markers for cardiovascular disease as well as new therapeutic targets. The successful accomplishment of this future research would contribute to increasing the standard of care for patients with cardiovascular disease, still representing the first cause of death worldwide.
